# Jumping patella is associated with inferior clinical outcomes after MPFL reconstruction for recurrent patellar dislocation: a retrospective cohort study

**DOI:** 10.1186/s43019-026-00347-z

**Published:** 2026-09-04

**Authors:** Daofeng Wang, Yang Liu, Zhengjie Tang, Zhijun Zhang, Hui Zhang

**Affiliations:** Sports Medicine Service, Beijing Jishuitan Hospital, Capital Medical University, Beijing, China

**Keywords:** Jumping patella, Knee torsion, Supratrochlear bump, Patellar maltracking, Recurrent patellar dislocation

## Abstract

**Background:**

To date, two studies have established the correlation between jumping sign and multiple bony abnormalities. However, no studies have yet reported the association between the jumping sign and clinical outcomes.

**Purpose:**

(1) To determine the bony risk factors of jumping patella and (2) to compare clinical outcomes between jumping and gliding patellae following medial patellofemoral ligament (MPFL) reconstruction.

**Patients and methods:**

Between 2018 and 2022, 85 patients with recurrent patellar dislocation (RPD) who underwent MPFL reconstruction were retrospectively reviewed. Patients were stratified into two groups based on the presence or absence of the preoperative patellar jumping sign. Standard hip-knee-ankle computed tomography (CT) scans were obtained for all patients, through which lower limb torsional deformities and bony morphology were evaluated. Bony risk factors for preoperative jumping patella were identified using logistic regression analysis. Clinical outcomes were compared between groups at final follow-up. Subgroup analyses were conducted to examine outcome differences between surgical procedures within each patellar tracking group.

**Results:**

This study included a total of 85 patients, with 36 cases in the jumping group. Multivariate regression analysis identified knee torsion (odds ratio [OR] = 7.16, 95% confidence interval [CI] = 1.64–31.33, *P* = 0.009) and supratrochlear bump (OR = 30.5, 95% CI = 6.95–133.77, *P* < 0.001) as significant factors associated with jumping patella. At the final follow-up, the jumping group demonstrated significantly lower Lysholm (75.7 versus 86.1, *P* = 0.002) and Kujala (80.8 versus 89.9, *P* = 0.003) scores compared with the gliding group. The jumping group showed significantly greater patellar medial laxity index (38% versus 22%, *P* = 0.001), and higher rates of residual maltracking (55.6% versus 16.3%, *P* < 0.001), jumping patella (27.8% versus 2%, *P* < 0.001), MPFL graft laxity (22.2% versus 4.1%, *P* = 0.01), and patellar redislocation (13.9% versus 2%, *P* = 0.035), compared with the gliding group. The subgroup analysis revealed that in cases of MPFL reconstruction combined with tibial tubercle transfer, the jumping patella had a much higher incidence of residual patellar maltracking (54.5% versus 15.6%, *P* = 0.005) and positive jumping sign (27.3% versus 0%, *P* = 0.002), compared with the gliding group, while no significant differences were found between the two groups in patient-reported outcome measures (PROMs), MPFL graft laxity, and patellar redislocation (all *P* > 0.05).

**Conclusions:**

Compared with the gliding patella, the jumping patella undergoing isolated MPFL reconstruction exhibited worse clinical outcomes. Although the combined procedure improved knee function and reduced redislocation rates to some extent, it could not significantly correct the persistent maltracking or jumping sign postoperatively.

**Supplementary information:**

The online version contains supplementary material available at https://doi.org/10.1186/s43019-026-00347-z.

## Introduction

Medial patellofemoral ligament (MPFL) reconstruction is the gold standard surgical procedure for recurrent patellar dislocation (RPD) requiring surgical intervention. Previous studies have shown that even with standardized procedures (such as graft selection and femoral tunnel position), the incidence of postoperative patellar redislocation remains between 2% and 8% [1, 2]. In the presence of multiple bony structural abnormalities, isolated MPFL reconstruction is insufficient to restore normal patellofemoral joint alignment and stress distribution [3], leading to poor postoperative outcomes such as persistent patellar maltracking and graft laxity [4]. These factors compromise knee function and patient satisfaction [5].

Clinically, certain patellae demonstrate a jumping phenomenon during dislocation, and this sign has been increasingly noticed and described in recent reports. Two studies have established correlations between this dynamic instability pattern and underlying bony abnormalities [6, 7], highlighting the need for special consideration of such cases, particularly when planning bony corrective procedures. Wang et al. found that the patients with a jumping sign exhibited rotational abnormalities consistent with high-grade J-sign in patients with RPD [7]. Recently, Dandu et al. conducted a multivariable analysis and found that the presence of a jumping sign is associated with magnetic resonance imaging (MRI) findings of relatively greater supratrochlear bump and knee torsion [6]. However, neither of the abovementioned studies explored the potential association between the jumping sign and clinical outcomes.

Therefore, the purposes of this study were: (1) to determine the bony risk factors of jumping patella; and (2) to compare clinical outcomes between patients demonstrating jumping versus gliding signs following MPFL reconstruction, with or without tibial tubercle transfer (TTT) for RPD. We hypothesized that the jumping sign is associated with bony structural abnormalities, and that this sign is related to poorer clinical outcomes following MPFL reconstruction.

## Patients and methods

### Patient selection

**Fig. 1:**
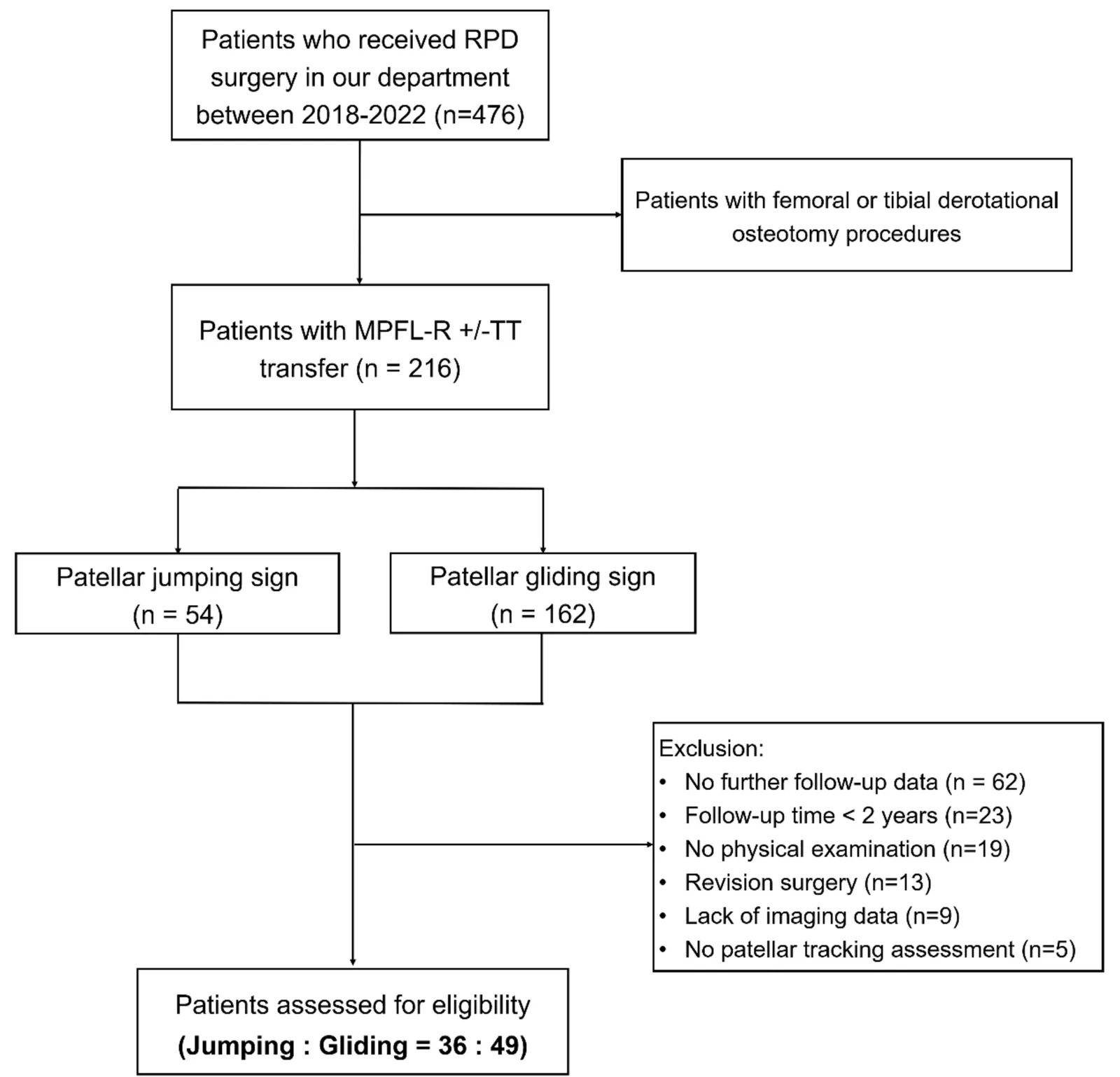
Flowchart of patient selection. Notably, most patients in the gliding group underwent isolated MPFL reconstruction (without concomitant TTT) and did not require hardware removal. Consequently, follow-up data were not obtainable for these patients, leading to considerable case loss

The study was initiated after ethical approval was obtained. All patients signed an informed consent form upon admission. Retrospective screening was conducted on patients who underwent surgical treatment for recurrent patellar dislocation at our institution from January 2018 to September 2022. All procedures were performed by a single, experienced surgeon specializing in sports medicine (Dr. XXX). Patients with the following criteria were included: (1) MPFL reconstruction, with or without TTT and trochleoplasty procedures; (2) postoperative follow-up data (> 2 years); (3) preoperative and postoperative documentation of patellar tracking; and (4) preoperative hip-knee-ankle scan in the standard position (patient supine with patella looking on the roof). Exclusion criteria included: (1) combined with femoral or tibial derotational osteotomy, (2) femoral tunnel malposition (the femoral tunnel placement deviating > 10 mm from the Schöttle point is defined as malposition in true lateral view of the knee [8]), (3) revision surgery, (4) unclear determination of preoperative patellar tracking, (5) follow-up duration < 2 years, and (6) generalized ligamentous laxity. The flowchart of patient selection is shown in Fig. 1.

### Patient grouping and study design

The quadrant method was used to assess the patellar J-sign grades [9]. This grading method was derived from the quadrant grading system [10] (Appendix 1). To reduce subjectivity, grades 1 and 2 were classified as low-grade J-sign in the present study. According to the description by Wang et al. [7], this study classified patients into jumping and gliding groups on the basis of preoperative patellar tracking (Appendix 2). The jumping sign was considered positive if a jumping phenomenon of the patella was observed during its dislocation or reduction process while the patient actively flexed and extended the knee.

The primary outcome measure of this study was the difference in patient-reported outcome measures (PROMs) between the two groups. The secondary outcomes were the incidences of residual patellar maltracking, MPFL graft laxity, and patellar redislocation rates between two signs.

### Surgical techniques

#### MPFL reconstruction

The MPFL was reconstructed using a semitendinosus tendon autograft (all patients). The harvested semitendinosus tendon measured approximately 20 cm in length and 6–7 mm in diameter after folding, which determined the femoral tunnel diameter. Based on the method described by Schöttle et al. [11], the femoral tunnel position was determined under intraoperative fluoroscopy. Two double-loaded suture anchors (Linvatec, USA) were inserted into the proximal one-third and equatorial regions of the patella, respectively. The graft was first fixed on the patellar side, and the free end of the graft was then pulled into the femoral tunnel and fixed with a bioabsorbable interference screw (Linvatec, USA) at 20–30° of knee flexion.

#### Additional procedures

Patients demonstrating a tibial tuberosity–trochlear groove (TT–TG) distance of ≥ 20 mm underwent Elmslie–Trillat medialization osteotomy for TT–TG normalization (target: 10–12 mm). Those with patella alta (Caton–Deschamps index ≥ 1.2) received additional tibial tubercle distalization to restore patellar height to 1.0. The transferred tibial tubercle was fixed with three cortical bone screws (DePuy Synthes, USA) [4]. Trochleoplasty was performed when intraoperative evaluation revealed persistent patellar maltracking during trochlear engagement after MPFL reconstruction with concomitant tibial tubercle osteotomy (TTO), if indicated. The trochlear groove was deepened via resection of the bony component beneath the trochlear cartilage, and the cartilage flap was then secured using two Versalok anchors (Linvatec, USA).

### Data collection

Age, sex, body mass index (BMI), affected side, time from initial dislocation to surgery, concomitant cartilage lesions, and number of dislocations were recorded for all patients preoperatively. The cartilage status (present or absent) was evaluated on the basis of arthroscopic findings at the time of initial surgery and at the final follow-up.

### Clinical outcomes

**Fig. 2:**
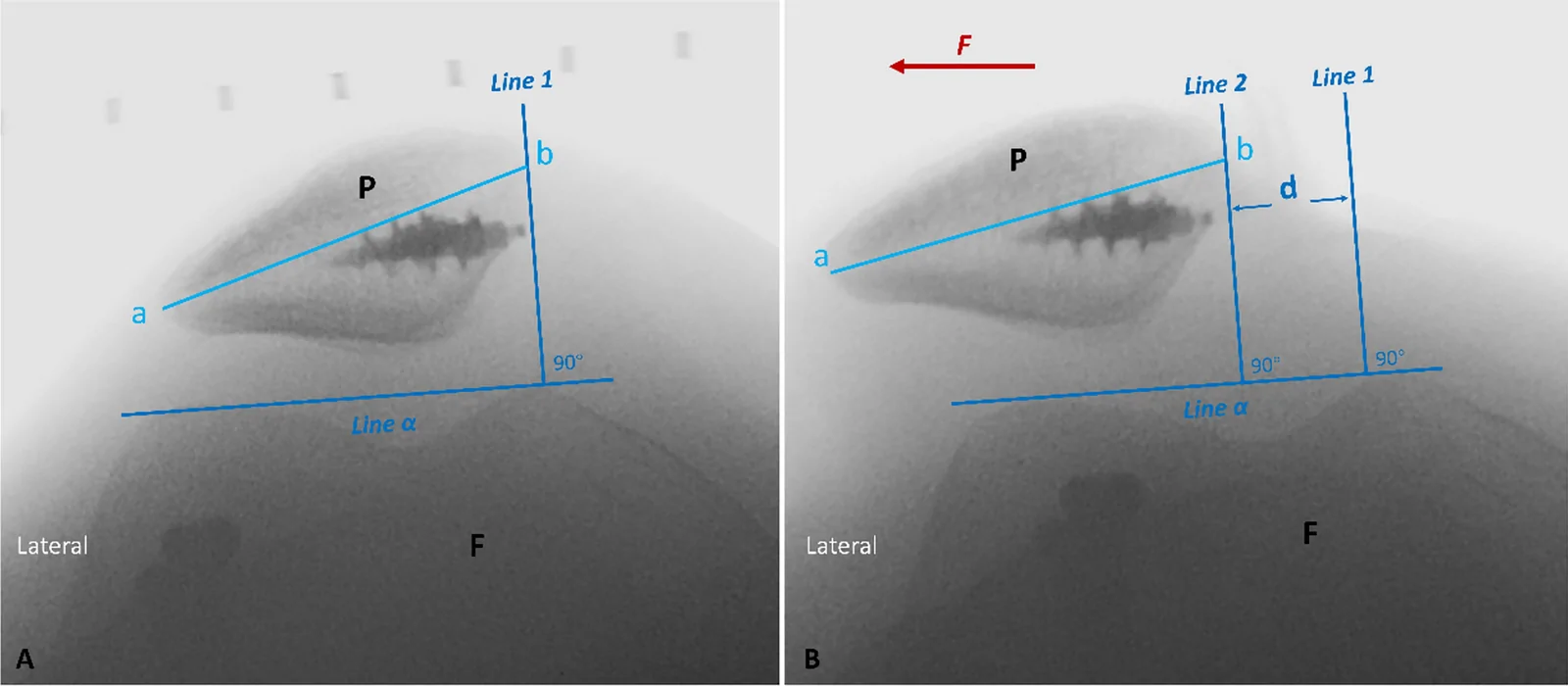
Quantitative evaluation of the patellar medial laxity index at final follow-up. (A) At 30° knee flexion in the neutral position, three key radiographic markers were established: (1) line ab representing the mediolateral patellar width, (2) line *α* drawn tangent to the femoral trochlear groove, and (3) line 1 perpendicular to *α* and intersecting the medial patellar border. (B) With 30° knee flexion under standardized lateral patellar stress (*F*), line 2 was similarly constructed perpendicular to *α* from the stressed medial patellar border. The MPFL laxity index (%) was then calculated by measuring the vertical displacement (d) between lines 1 and 2 relative to patellar width (d/ab × 100%). P, patella; F, femur

The objective evaluation included preoperative and postoperative patellar maltracking (J-sign or jumping patella), fluoroscopic assessment of MPFL graft laxity, and postoperative patellar redislocation events (clinical failure). According to the method described by Zhang et al., another examiner (XXX) placed the thumb on the medial border of the patella and the other four fingers on the posterior margin of the lateral femoral condyle, then applied maximum thumb force to exert a lateral translation stress, followed by axial fluoroscopic evaluation of MPFL graft laxity index [4]. As shown in Fig. 2, the ratio of patellar displacement relative to the trochlea (d) to the mediolateral patellar width (ab) after stress application, expressed as a percentage, represents the MPFL graft laxity index. Residual graft laxity was defined as the patellar medial laxity index > 50% (Fig. 2).

### Bony structures evaluation

**Fig. 3:**
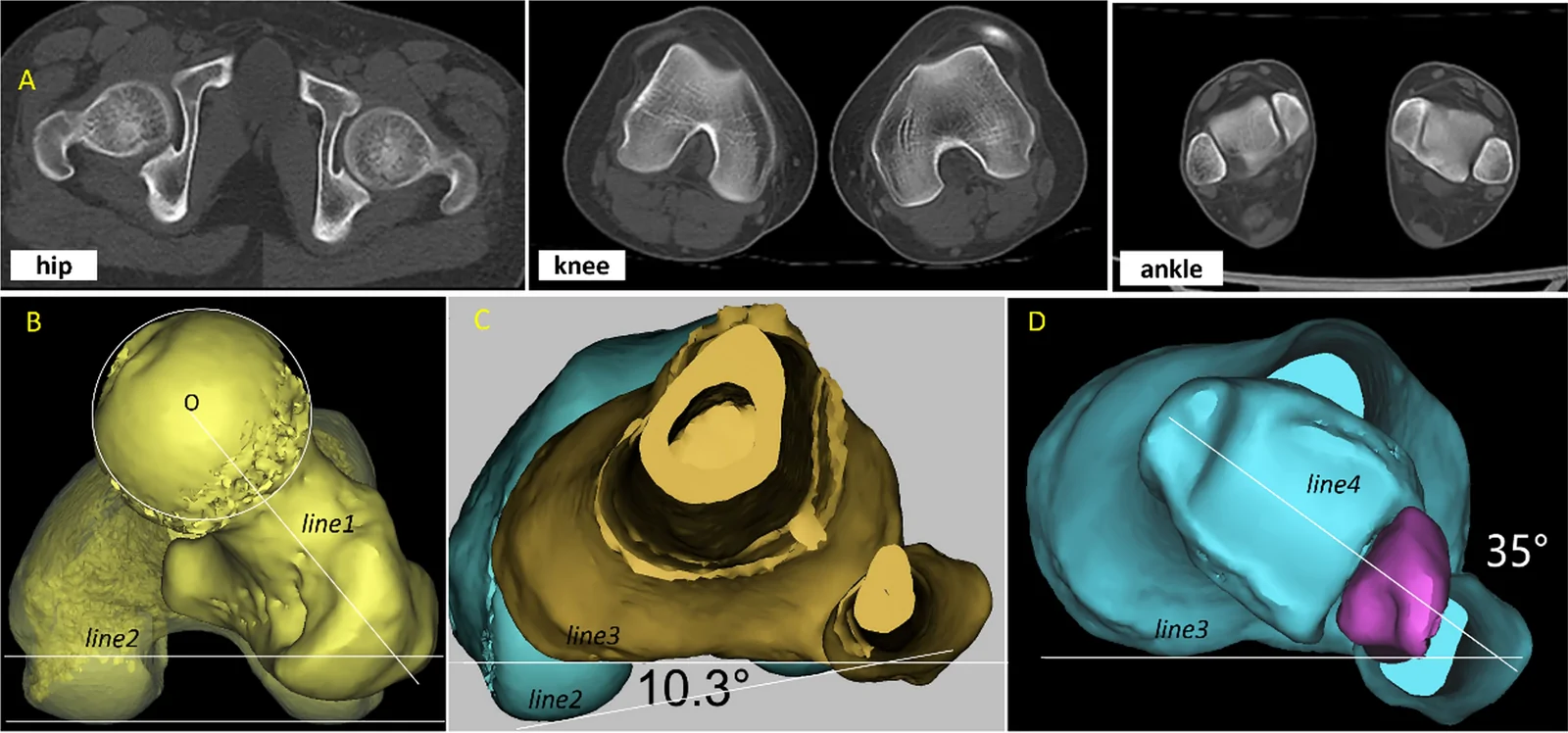
Standard CT scan of the hip-knee-ankle and measurements of (A) lower limb torsion parameters, (B) femoral anteversion, (C) knee torsion, and (D) tibial torsion

Standard hip-knee-ankle computed tomography (CT) scans (Fig. 3A) were conducted preoperatively for all patients. Three-dimensional (3D) reconstruction of the lower limb bones was carried out using Mimics 20.0. After three-dimensional reconstruction, the bone models were obtained. The 3D models were then rotated and translated in Mimics to obtain the measurement models for each parameter, followed by two-dimensional measurements in Photoshop [9, 12]. The specific methods are shown in Fig. 3 below (Fig. 3).

#### Femoral anteversion angle (FAA)

The center points of the femoral head and neck were identified through a circle-fitting method. The FAA is defined as the angle formed between line 1, which connects the femoral head and neck, and line 2, which links the posterior condyles of the femur (Fig. 3B). FAA > 30° was considered abnormal [13].

#### Knee torsion

For knee rotation measurement, the reconstructed tibia was used to establish the posterior–anterior view, after which the model was adjusted to align the posterior edge of the tibial plateau horizontally. Knee torsion was defined as the angle between the posterior tibial plateau tangent (line 3) and line 2 (Fig. 3C). Knee torsion > 12° was considered abnormal [14, 15].

#### Tibial torsion

This measurement relies on the rotational angle of the proximal tibia in relation to the distal tibia. The posterior tibial plateau was kept horizontally aligned. The center point of the distal tibia and fibula was identified, and line 4 was drawn accordingly. Tibial torsion angle was defined as the angle between line 3 and line 4 (Fig. 3D). Tibial torsion > 30° was considered abnormal [16].

#### Tibial tubercle malposition

The tibial tuberosity–trochlear groove (TTTG) distance was determined by superimposing the trochlear level and tibial tuberosity level on the CT cross-sectional image (Roman arch appearance of the intercondylar notch). Based on the method described by Seitlinger et al. [17], the tibial tubercle–posterior cruciate ligament (TT–PCL) distance is the mediolateral distance between the tip of the tibial tubercle and the medial margin of the PCL, parallel to the tibial posterior condylar line. TTTG > 20 mm [18] or TT–PCL > 24 mm [19] were considered abnormal, respectively.

#### Trochlear dysplasia

Trochlear dysplasia was evaluated based on the Dejour classification method using the true lateral view of the knee. Severe trochlear dysplasia was defined as types B and D [18].

#### Trochlear and patellar-relative parameters

This study evaluated trochlear morphological parameters, including the supratrochlear bump, lateral trochlear inclination (LTI), sulcus angle, and trochlear depth according to the method described by Wang et al. [7]. Supratrochlear bump > 5 mm, LTI ≤ 12°, sulcus angle > 145°, and trochlear depth ≤ 3 mm were considered abnormal [7]. The Caton–Deschamps index (CDI) is commonly used to evaluate patellar height. It can be measured on the lateral view at 30° of knee [7]. The bisect offset index, patellar lateral tilt angle, and patella–trochlear groove distance were used to measure preoperative and postoperative patellar alignment according to the method described by Xue et al. [20].

### Patient-reported outcomes measures

The preoperative and postoperative Lysholm score, Kujala score, and Tegner score were assessed for all patients. All evaluations were conducted in the outpatient clinic. Questionnaires were distributed to patients and collected upon confirmation of complete responses.

### Second-look arthroscopic findings

For patients who underwent TTT, if removal of the internal fixation was requested, the procedure was considered only after at least 2 years postoperatively. In these cases, we routinely performed a second-look arthroscopic evaluation during hardware removal to assess concomitant lesions, such as cartilage injury.

### Sample size

This study used the Lysholm score as the basis for sample size calculation. In the pilot study, 20 patients in the jumping patella group and 20 patients in the gliding patella group were evaluated. At the final follow-up, the mean Lysholm scores (standard deviation, SD) were 76.2 (13.4) and 86.4 (12.3) in the jumping and gliding groups, respectively. Using G*Power software (Version 3.1, Franz Paul), the effect size for evaluating intergroup comparisons was calculated as 0.79. With α set at 0.05 and power (1−*β*) at 0.80, the minimum required sample size was determined to be 21 cases per group to detect clinically significant differences in functional scores between groups.

### Statistical analysis

All quantitative data were tested for normality. Normally distributed variables were expressed as mean ± standard deviation, whereas non-normal data were reported as median (interquartile range). Categorical variables were summarized as frequencies (percentages).

The intraclass correlation coefficient (ICC) was computed to evaluate the reliability of the measurement method used in this study. Sample size calculation for intraclass correlation analysis was performed using PASS 2021 (NCSS, LLC, Kaysville, UT) [21, 22]. With approximately 90% confidence, it was determined that a stable consistency coefficient could be achieved with just 14 samples when two measurers independently assessed the same parameters using the same approach. Consequently, 20 samples were randomly selected. Two experienced researchers (XXX and XXX) independently measured the lower limb bony structure parameters, and along with the existing measurement results, the intraclass correlation coefficients were calculated.

The differences in quantitative variables between groups were analyzed using independent sample *t*-tests or Wilcoxon rank-sum tests, while differences in categorical variables were analyzed using Chi-square tests or Fisher’s exact tests. Receiver operating characteristic (ROC) curve analysis was performed to evaluate the predictive value of bony structures for the jumping patella. Binary logistic regression was used to identify bony risk factors associated with preoperative jumping sign. All statistical analyses were conducted using *R* (version 4.2.1) and SPSS (version 26.0; IBM Corp), with the significance threshold set at *P* < 0.05.

## Results

### Patient characteristics

A total of 85 patients (85 knees) were included, with 36 showing the jumping sign. A total of 30 (35.3%) patients underwent isolated MPFL reconstruction, 51 (60%) cases received combined tibial tubercle transfer procedure, and 4 (4.7%) knees underwent combined trochleoplasty. In the gliding group, all three patients who underwent trochleoplasty also received concomitant TTO. All patients were followed up for more than 2 years. The two groups were well comparable in terms of age, sex, number of dislocations, disease duration, preoperative knee function scores, surgical procedures, cartilage injury, and follow-up duration (*P* > 0.05). Patient characteristics are summarized in Table 1.

**Table 1:** Basic characteristics between the two signs

Variables	Jumping sign (*n* = 36)	Gliding sign (*n* = 49)	*P* value
Age, years	21.4 ± 5.4	23 ± 7.1	0.272
Sex (female)	28 (77.8%)	39 (79.6%)	0.840
Side (left)	23 (63.9%)	26 (53.1%)	0.318
No. of dislocations			
> 1, ≤ 2	15 (41.7%)	30 (61.2%)	0.195
> 2, ≤ 5	12 (33.3%)	10 (20.4%)	
> 5	9 (25%)	9 (18.4%)	
Duration, months	36 (3-60)	48 (12-96)	0.094*
PROMs (presurgery)			
Lysholm score	53.4 ± 9.8	56.8 ± 10.6	0.189
Tegner score	2.8 ± 0.7	3.1 ± 0.9	0.324
Kujala score	52.9 ± 10.4	53.7 ± 11.2	0.842
Procedures			
MPFL-R alone	13 (36.1%)	17 (34.7%)	0.746
with TTM alone	10 (27.8%)	18 (36.7%)	
with TTD alone	4 (11.1%)	4 (8.2%)	
with TTM+TTD	8 (22.2%)	7 (14.3%)	
with trochleoplasty	1 (2.2%)	3 (6.1%)	
Cartilage injury (+)	10 (27.8%)	12 (24.5%)	0.732
Follow-up, years	3.6 ± 1.4	3.9 ± 1.6	0.121

^*^Wilcoxon rank-sum tests

PROMs, patient-reported outcome measures; MPFL-R, medial patellofemoral ligament reconstruction; TTM, tibial tubercle medialization; TTD, tibial tubercle distalization

### Reliability and accuracy of measurements

The interobserver intraclass correlation coefficients for bony structural parameter measurements were all > 0.80, confirming that the assessment methods used in this study have high reproducibility. See Appendix 3 for more reliability analysis details.

### Bony risk factors of preoperative jumping patella

Patients with the jumping sign exhibited a flatter trochlea, greater knee torsion (12.2° versus 7.8°, *P* < 0.001), higher supratrochlear bump (6.2 mm versus 3.4 mm, *P* < 0.001), and increased sulcus angle (152.5° versus 146.7°, *P* = 0.001). The jumping group also showed significantly higher proportions of abnormal knee torsion (52.8% versus 16.3%, *P* < 0.001) and supratrochlear bump (80.6% versus 20.4%, *P* < 0.001), compared with the gliding group. No significant differences were found between the two groups in femoral anteversion, tibial torsion, trochlear dysplasia, TT–TG, TT–PCL, or patellar height (*P* > 0.05) (Table 2). Univariate regression analysis confirmed these findings. Multivariate regression analysis demonstrated that excessive knee torsion (OR = 7.16, 95% CI = 1.64–31.33, *P* = 0.009) and supratrochlear bump (OR = 30.5, 95% CI = 6.95–133.77, *P* < 0.001) were significant contributing factors to the formation of jumping patella (Table 3). In addition, ROC curve analysis demonstrated that knee torsion (AUC = 0.73) and supratrochlear bump (AUC = 0.86) showed the strongest correlation with jumping patella, while the remaining parameters exhibited poor diagnostic performance (all AUC < 0.66) (Appendix 4).

**Table 2:** Preoperative radiologic characteristics between the two signs

Variables	Jumping sign (*n* = 36)	Gliding sign (*n* = 49)	*P* value
Femoral anteversion, °	23.8 ± 10.8	21.1 ± 8.6	0.208
> 30	9 (25%)	5 (10.2%)	0.069
Knee torsion, °	12.2 ± 5.9	7.8 ± 4.6	** < 0.001**
> 12°	19 (52.8%)	8 (16.3%)	< 0.001
Tibial torsion, °	26.8 ± 10	26.4 ± 7.8	0.844
> 30°	13 (36%)	13 (26.5%)	0.344
Trochlear dysplasia	31 (86%)	37 (75.5%)	0.227
Supratrochlear bump, mm	6.2 ± 1.6	3.4 ± 2.0	**< 0.001**
> 5 mm	29 (80.6%)	10 (20.4%)	< 0.001
Sulcus angle, °	152.5 ± 8.8	146.7 ± 6.5	**0.001**
> 145°	29 (80.6%)	29 (59.2%)	0.037
Lateral trochlear inclination, °	13 ± 4.2	14.5 ± 5	0.133
< 12°	18 (50%)	16 (32.7%)	0.107
Trochlear depth, mm	2.5 ± 1.1	3.2 ± 1.3	**0.012**
< 3 mm	26 (72.2%)	20 (40.8%)	0.004
TT–TG, mm	21.1 ± 3.4	19.7 ± 3.7	0.069
> 20 mm	24 (66.7%)	24 (49%)	0.104
TT–PCL, mm	22 ± 2.9	22 ± 3.1	0.973
> 24 mm	8 (22.2%)	12 (24.5%)	0.808
CDI	1.2 ± 0.3	1.1 ± 0.2	0.532
> 1.2	13 (36%)	16 (32.7%)	0.740

TT–TG, tibial tuberosity–trochlear groove; TT–PCL, tibial tubercle–posterior cruciate ligament; CDI, Caton–Deschamps index

**Table 3:** Logistic analysis of bony risk factors contributing to preoperative jumping patella

Bony structures	Knees	Univariate analysis		Multivariate analysis	
		Odds ratio (95% CI)	*P* value	Odds ratio (95% CI)	*P* value
Knee torsion, °	85				
≤ 12	27	Reference		Reference	
> 12	58	5.73 (2.11–15.59)	**< 0.001**	7.16 (1.64–31.33)	**0.009**
Supratrochlear bump, mm	85				
≤ 5	39	Reference		Reference	
> 5	46	16.16 (5.49–47.52)	**< 0.001**	30.50 (6.95–133.77)	**< 0.001**
Sulcus angle, °	85				
≤ 145	58	Reference		Reference	
> 145	27	2.86 (1.05–7.79)	**0.040**	3.12 (0.67–14.48)	0.146
Trochlear depth, mm	85				
> 3	46	Reference		Reference	
≤ 3	39	3.77 (1.49–9.51)	**0.005**	2.61 (0.70–9.69)	0.152

### Clinical outcomes between the two signs

At the final follow-up, the jumping group demonstrated significantly lower Lysholm (75.7 versus 86.1, *P* = 0.002) and Kujala scores (80.8 versus 89.9, *P* = 0.003) compared with the gliding group, while no significant difference was observed in Tegner scores between the two groups (3.8 versus 4.4, *P* = 0.18). Among all patients, 28 (32.9%) patients demonstrated persistent patellar maltracking, 11 (12.9%) cases had jumping patella, 10 (11.8%) cases showed graft laxity, and 6 (7.1%) patients experienced patellar redislocation. The jumping group showed significantly greater patellar medial laxity index (38% versus 22%, *P* = 0.001), and higher rates of residual maltracking (55.6% versus 16.3%, *P* < 0.001), jumping patella (27.8% versus 2%, *P* < 0.001), MPFL graft laxity (22.2% versus 4.1%, *P* = 0.01), and patellar redislocation (13.9% versus 2%, *P* = 0.035), compared with the gliding group (Table 4). In addition, the jumping group also showed greater postoperative lateral patellar translation compared with the gliding group. No significant differences were observed in cartilage injury between the two groups. One patient in the gliding group presented with a jumping sign postoperatively (Table 4).

**Table 4:** Comparisons of final follow-up outcomes between the two groups

Outcomes	Variables	Jumping group (*n* = 36)	Gliding group (*n* = 49)	*P* value
Patient-reported outcome measures	Lysholm score	75.7 ± 13.9	86.1 ± 11.1	0.002
	Tegner score	3.8 ± 1.5	4.4 ± 1.9	0.180
	Kujala score	80.8 ± 12.7	89.9 ± 9.7	0.003
Physical examinations	Residual J-sign (−)	16 (44.4%)	41 (83.7%)	< 0.001
	1+	14 (28.9%)	7 (14.3%)	
	2+	6 (16.7%)	1 (2.0%)	
	Jumping sign (+)	10 (27.8%)	1 (2.0%)	< 0.001
	Patellar medial laxity index, %	38 ± 24	22 ± 13	0.001
	MPFL graft residual laxity (+)	8 (22.2%)	2 (4.1%)	0.010
	Patellar redislocation (+)	5 (13.9%)	1 (2%)	0.035
Radiographic findings	Patellar bisect offset index, %	104.1 ± 42	88.3 ± 12.8	0.039
	Patellar lateral tilt, °	30.1 ± 16.3	22.6 ± 7.1	0.018
	Patella–trochlear groove, mm	1.6 ± 6.1	−4.7 ± 5.3	0.005
Second-look arthroscopic findings*	Combined cartilage injury (+)	3 (8.3%)	5 (10.2%)	0.770

^*^For patients who underwent combined tibial tubercle transfer only

The subgroup analysis based on the procedure revealed that among cases undergoing isolated MPFL reconstruction, the difference in clinical outcomes between the two groups was highly consistent with that observed in the overall cohort. In cases of MPFL reconstruction combined with TTT, the jumping patella also had a significantly higher incidence of residual patellar maltracking (54.5% versus 15.6%, *P* = 0.005) and positive jumping sign (27.3% versus 0%, *P* = 0.002) compared with the gliding group, while no significant differences were found between the two groups in PROMs, MPFL graft laxity, patellar redislocation, or lateral patellar translation (all *P* > 0.05) (Table 5).

**Table 5:** Procedure-based subgroup analysis: comparing clinical outcomes between the two signs under different surgical techniques

	MPFL reconstruction alone (*n* = 31)			MPFL reconstruction with TTT (*n* = 54)		
	Jumping sign (*n* = 14)	Gliding sign (*n* = 17)	*P* value	Jumping sign (*n* = 22)	Gliding sign (*n* = 32)	*P* value
Lysholm score	65.5 ± 11.6	76.7 ± 12.3	**0.004**	84 ± 9.4	87.1 ± 9.9	0.319
Tegner score	3.5 ± 1.9	3.3 ± 1.5	0.877	3.9 ± 1.4	4.5 ± 1.9	0.302
Kujala score	70.1 ± 9.7	79.3 ± 11.2	**0.002**	89.4 ± 6.7	91 ± 8.4	0.528
Residual J-sign (−)	6 (42.9%)	14 (82.4%)	0.073	10 (45.5%)	27 (84.4%)	**0.005**
1+	5 (35.7%)	2 (11.8%)		9 (40.9%)	5 (15.6%)	
2+	3 (21.4%)	1 (5.9%)		3 (13.6%)	0	
Jumping sign (+)	4 (28.6%)	1 (5.9%)	0.087	6 (27.3%)	0	**0.002**
Patellar medial laxity index, %	52 ± 28.8	23 ± 15.4	**0.003**	28 ± 11.7	21 ± 11.3	**0.050**
MPFL graft residual laxity (+)	7 (50%)	1 (5.9%)	**0.005**	1 (4.5%)	1 (3.1%)	0.788*
Patellar redislocation (+)	4 (28.6%)	1 (5.9%)	0.087	1 (4.5%)	0	0.177*
Patellar bisect offset index, %	119.4 ± 57.7	92.5 ± 20	0.252	93.1 ± 19.8	87.3 ± 10.5	0.202
Patellar lateral tilt, °	38.6 ± 17.7	24.1 ± 9.8	0.017	23.8 ± 12.2	22.2 ± 7.2	0.599
Patella–trochlear groove, mm	0.7 ± 1.2	−0.4 ± 0.8	0.065	−0.2 ± 0.8	−0.5 ± 0.5	0.123

^*^Fisher’s Exact test

MPFL, medial patellofemoral ligament; TTT, tibial tubercle transfer

The subgroup analysis based on postoperative jumping sign revealed that patients with residual jumping sign had lower Lysholm (71.6 versus 82.3, *P* = 0.019) and Kujala scores (76.1 versus 86.6, *P* = 0.011), higher rates of MPFL graft laxity (54.5% versus 5.4%, *P* < 0.001) and failure rate (45.5% versus 1.4%, *P* < 0.001) than those with normal postoperative tracking (Table 6).

**Table 6:** Postoperative jumping sign-based subgroup analysis: exploring the direct association between residual jumping sign and clinical outcomes

Variables	With residual jumping sign (*n* = 11)	Without residual jumping sign (*n* = 74)	*P* value
Lysholm score	71.6 ± 16.0	82.3 ± 12.6	0.019
Tegner score	3.5 ± 2.0	4.2 ± 1.7	0.702
Kujala score	76.1 ± 13.1	86.6 ± 11.7	0.011
Patellar medial laxity index, %	58 ± 29.9	24 ± 12.6	0.003
MPFL graft residual laxity (+)	6 (54.5%)	4 (5.4%)	< 0.001
Patellar redislocation (+)	5 (45.5%)	1 (1.4%)	< 0.001*
Patellar bisect offset index, %	122.9 ± 49.7	90.6 ± 22.9	0.002
Patellar lateral tilt, °	39.5 ± 16.9	23.4 ± 10	0.011
Patella–trochlear groove, mm	0.9 ± 0.8	–0.4 ± 0.7	< 0.001
Combined cartilage injury (+)	1 (9.1%)	7 (9.5%)	0.969

^*^Fisher’s Exact test

## Discussion

The most important findings of this study were that (1) excessive knee torsion and the supratrochlear bump were significantly associated with the formation of preoperative jumping patella; and (2) compared with gliding patella, patients with jumping patella who underwent isolated MPFL reconstruction exhibited worse clinical outcomes. Furthermore, while the addition of TTO can further improve subjective functional scores and patellar instability, its effect on persistent postoperative maltracking remains limited.

Wang et al. found that knees with the jumping sign exhibited a higher proportion of increased FAA (40.8% versus 24.4%), excessive knee torsion (61.2% versus 15.3%), pronounced supratrochlear bump (73.1% versus 32.3%), and flatter lateral trochlear inclination (81.3% versus 27.5%) [7]. These proportions indicated that patients with a jumping J-sign have more complex bony structural abnormalities. Another study suggested that the trochlear bump may contribute to the pathogenesis of this sign [6]. The present study also confirms that excessive knee torsion and supratrochlear bump played a significant role in the formation of a jumping patella. Effectively minimizing residual maltracking and positive jumping sign contributes to significant improvements in knee functional outcomes. Therefore, we recommend routine hip-knee-ankle CT scans with 3D assessment for patients with RPD, particularly those with a jumping J-sign, to identify the specific bony deformities, crucial for surgical planning.

MPFL reconstruction is the basic intervention for patients with RPD that require surgical management [23–25]. Clinically, additional procedures may be performed to maintain the patellofemoral joint congruence and patellar tracking. Combined tibial tubercle osteotomy is frequently employed in cases with increased TT–TG distance, and this technique has been demonstrated to effectively reduce patellofemoral joint contact pressure and improve patellofemoral congruence [26, 27]. For patients with excessive femoral anteversion and high-grade patellar maltracking, distal derotational femoral osteotomy (DDFO) has shown superior clinical and radiological outcomes, although it is associated with greater surgical trauma [4, 28]. Trochleoplasty can effectively correct pathologically prominent trochlear bump and improve knee stability in patients with high-grade trochlear dysplasia [29, 30]. However, there is no consensus regarding the management of excessive knee torsion. A recent study reported that knee torsion showed no significant changes following DDFO (10.2° versus 9.4°, *P* = not significant) [31]. However, Zhang et al. [32] reported in a series of 49 cases that DDFO could correct the knee torsion by approximately 5° (preoperative versus postoperative: 17° versus 12°, *P* < 0.001), with a mean follow-up of 3.8 years. Given that knee torsion is primarily a soft tissue-dependent deformity, achieving its full correction may require a longer time. Consequently, differences in the timing of measurement (e.g., immediately postoperatively versus 2 years postoperatively) could lead to significant variations, which may explain the discrepancies between the two studies. Further clinical data are required to determine the precise effect of DDFO on knee torsion.

This study found that patients with jumping patella had a significantly higher incidence of residual patellar maltracking and positive jumping sign postoperatively compared with those with gliding patella, regardless of whether MPFL reconstruction or combined TTT was performed. Dennis et al. demonstrated that persistent J-sign postoperatively is associated with poorer long-term clinical outcomes and return-to-sport levels [5]. Our findings further indicated that jumping patella treated with MPFL reconstruction alone had a higher rate of postoperative patellar redislocation, and significantly worse functional outcomes. Although combined TTT significantly reduced graft laxity and improved subjective functional scores, it still failed to effectively control the occurrence of persistent patellar maltracking and jumping sign. This suggests that the occurrence of jumping patella may not be influenced by tibial tubercle malposition. Analysis of bony structural risk factors also confirmed this finding.

Another significant finding of this study is that MPFL reconstruction combined with TTO effectively improves subjective functional scores. In the surgical management of recurrent patellar instability, the decision to perform concomitant TTO does not depend solely on the degree of tibial tubercle lateralization. Recently, Dennis et al. reported that MPFL reconstruction combined with TTO effectively improves clinical outcomes at 2 years postoperatively in revision cases, with clinical benefits comparable to those of primary procedures [33]. Their indications for TTO primarily involved chondral defects (off-loadable), a jumping J-sign, or anterior knee pain. Their findings support our observations. In patients presenting with a jumping J-sign, TTO may be considered regardless of the TT–TG distance.

One patient (female, 20 years old) in the gliding group developed jumping sign postoperatively. Preoperatively, this patient exhibited several pronounced structural osseous abnormalities, including moderate femoral anteversion (FAA = 28°), substantial knee torsion (19°), trochlear dysplasia (bump = 5.4 mm, sulcus angle = 152°), lateralization of the tibial tubercle (TT–TG = 22 mm), and patella alta (CDI = 1.3). Preoperatively, combined distal femoral derotational osteotomy and tibial tubercle osteotomy were recommended for this patient, but the patient opted for isolated MPFL reconstruction. Despite favorable postoperative patellar stability, we hypothesize that the postoperative worsening of patellar tracking might be related to the unresolved underlying osseous deformities.

The primary challenge of current patellar tracking classifications is to identify which pathological tracking patterns require additional procedures beyond MPFL reconstruction. A recent reliability study reported low interobserver agreement for the quadrant method (kappa = 0.51, *P* < 0.60) [34]. This suggests that although individual observers maintain consistent standards when assessing patellar tracking, reproducibility between different observers is relatively poor, limiting the generalizability of treatment strategies based on these classification systems. The present study found that classifying patellar tracking using the jumping sign and gliding sign achieved higher interobserver agreement (kappa = 0.88). This result is consistent with previous research [7]. Furthermore, this classification system encompasses a broader range of cases and provides more consistent treatment strategies, highlighting its clinical utility.

This study has limitations. This study employed a retrospective cohort design. In the gliding group, the majority of patients underwent isolated MPFL reconstruction. Since these patients did not require hardware removal, nearly one-third failed to return for outpatient follow-up. Although the included cohort met the sample size requirements, the relatively high loss to follow-up rate may have influenced the study findings to some extent. In addition, the small number of patients who underwent trochleoplasty (*n* = 4) precluded a meaningful subgroup analysis, which limits the generalizability of our conclusions. Finally, despite all patients having a minimum follow-up of 2 years, the overall follow-up duration remained relatively short. Consequently, the potential association between persistent patellar maltracking or positive jumping sign and long-term clinical outcomes could not be definitively established.

## Conclusions

Excessive knee torsion and the supratrochlear bump were significantly associated with the formation of preoperative jumping patella. Compared with the gliding patella, the jumping patella undergoing isolated MPFL reconstruction exhibited worse PROMs, a higher incidence of residual MPFL graft laxity, and patellar redislocation. These findings support the research hypothesis. While the addition of TTO can further improve subjective functional scores and patellar instability, its effect on persistent postoperative maltracking remains limited.

## Supplementary information

Supplementary material 1

Supplementary material 2

Supplementary material 3

Supplementary material 4

## Data Availability

No datasets were generated or analyzed during the current study.

## Abbreviations

PROMs: patient-reported outcome measures
MPFL-R: medial patellofemoral ligament reconstruction
TTM: tibial tubercle medialization
TTD: tibial tubercle distalization
TT–TG: tibial tuberosity–trochlear groove
TT–PCL: tibial tubercle–posterior cruciate ligament
CDI: Caton–Deschamps index

## References

[1] Smith TO, Walker J, Russell N (2007) Outcomes of medial patellofemoral ligament reconstruction for patellar instability: a systematic review. Knee Surg Sports Traumatol Arthrosc 15:1301–1314. 10.1007/s00167-007-0390-0 17684729

[2] Testa EA, Camathias C, Amsler F, Henle P, Friederich NF, Hirschmann MT (2017) Surgical treatment of patellofemoral instability using trochleoplasty or MPFL reconstruction: a systematic review. Knee Surg Sports Traumatol Arthrosc 25:2309–2320. 10.1007/s00167-015-3698-1 26187008

[3] Kaiser P, Schmoelz W, Schöttle PB, Heinrichs C, Zwierzina M, Attal R (2019) Isolated medial patellofemoral ligament reconstruction for patella instability is insufficient for higher degrees of internal femoral torsion. Knee Surg Sports Traumatol Arthrosc 27:758–765. 10.1007/s00167-018-5065-5 30062643

[4] Zhang Z, Song G, Li Y, Zheng T, Ni Q, Feng H et al. (2021) Medial patellofemoral ligament reconstruction with or without derotational distal femoral osteotomy in treating recurrent patellar dislocation with increased femoral anteversion: a retrospective comparative study. Am J Sports Med 49:200–206. 10.1177/0363546520968566 33180556

[5] Dennis ER, Ammerman BM, Nguyen JT, Marmor WA, Pahapill NK, Propp BE et al. (2025) Outcomes after isolated medial patellofemoral ligament reconstruction for recurrent patellar instability: influence of persistent postoperative apprehension and J-sign. Am J Sports Med 53:1931–1939. 10.1177/03635465251339822 40478223

[6] Dandu N, Hevesi M, Phillips AR, Haneberg EC, Elias TJ, Wang Z et al. (2025) Anatomic drivers of J-sign presence and severity: if there is a jump, look for a bump. Am J Sports Med. 10.1177/03635465251322788 40071349

[7] Wang D, Liu Y, Sun J, Fu Q, Lv C, Yue T et al. (2025) Patients with jumping sign exhibit rotational and bony structural abnormalities consistent with high-grade J-sign in recurrent patellar dislocation. Knee Surg Sports Traumatol Arthrosc. 10.1002/ksa.12584 39803705

[8] Servien E, Fritsch B, Lustig S, Demey G, Debarge R, Lapra C et al. (2011) In vivo positioning analysis of medial patellofemoral ligament reconstruction. Am J Sports Med 39:134–139. 10.1177/0363546510381362 20929935

[9] Wang D, Zhang Z, Cao Y, Song G, Zheng T, Di M et al. (2024) Recurrent patellar dislocation patients with high-grade J-sign have multiple structural bone abnormalities in the lower limbs. Knee Surg Sports Traumatol Arthrosc 32:1650–1659. 10.1002/ksa.12186 38651601

[10] Rousseau-Saine A, Nault ML, Hiemstra LA (2023) What is the J-sign and why is it important? Curr Opin Pediatr 35:97–101. 10.1097/mop.0000000000001193 36592028

[11] Schöttle PB, Schmeling A, Rosenstiel N, Weiler A (2007) Radiographic landmarks for femoral tunnel placement in medial patellofemoral ligament reconstruction. Am J Sports Med 35:801–804. 10.1177/0363546506296415 17267773

[12] Takagi S, Sato T, Watanabe S, Tanifuji O, Mochizuki T, Omori G et al. (2018) Alignment in the transverse plane, but not sagittal or coronal plane, affects the risk of recurrent patella dislocation. Knee Surg Sports Traumatol Arthrosc 26:2891–2898. 10.1007/s00167-017-4806-1 29150745

[13] Zhang Z, Zhang H, Song G, Zheng T, Ni Q, Feng H (2020) Increased femoral anteversion is associated with inferior clinical outcomes after MPFL reconstruction and combined tibial tubercle osteotomy for the treatment of recurrent patellar instability. Knee Surg Sports Traumatol Arthrosc 28:2261–2269. 10.1007/s00167-019-05818-3 31797022

[14] Wang D, Cao Y, Zhang Z, Zheng T, Wang X, Zhang H (2025) Knee torsion predicts the preoperative J-sign grade in patients with recurrent patellar dislocation: a prospective study. Bone Joint J 107-b:504–513. 10.1302/0301-620x.107b5.Bjj-2024-0766.R2 40306653

[15] Zhang ZJ, Dimeng LQ, Cao YW, Zheng T, Song GY, Li Y et al. (2022) Predictors of graft failure after primary medial patellofemoral ligament reconstruction. Orthop J Sports Med 10:23259671221138856. 10.1177/23259671221138854 36532153 PMC9747879

[16] Zhang Z, Zhang H, Song G, Zheng T, Feng H (2020) A pre-operative grade 3 J-sign adversely affects short-term clinical outcome and is more likely to yield MPFL residual graft laxity in recurrent patellar dislocation. Knee Surg Sports Traumatol Arthrosc 28:2147–2156. 10.1007/s00167-019-05736-4 31612265

[17] Seitlinger G, Scheurecker G, Högler R, Labey L, Innocenti B, Hofmann S (2012) Tibial tubercle-posterior cruciate ligament distance: a new measurement to define the position of the tibial tubercle in patients with patellar dislocation. Am J Sports Med 40:1119–1125. 10.1177/0363546512438762 22415209

[18] Dejour H, Walch G, Nove-Josserand L, Guier C (1994) Factors of patellar instability: an anatomic radiographic study. Knee Surg Sports Traumatol Arthrosc 2:19–26. 10.1007/bf01552649 7584171

[19] Tensho K, Shimodaira H, Akaoka Y, Koyama S, Hatanaka D, Ikegami S et al. (2018) Lateralization of the tibial tubercle in recurrent patellar dislocation: verification using multiple methods to evaluate the tibial tubercle. J Bone Joint Surg Am 100:e58. 10.2106/jbjs.17.00863 29715229

[20] Xue Z, Song GY, Liu X, Zhang H, Wu G, Qian Y et al. (2018) Excessive lateral patellar translation on axial computed tomography indicates positive patellar J sign. Knee Surg Sports Traumatol Arthrosc 26:3620–3625. 10.1007/s00167-018-4897-3 29560511

[21] Wang D, Li J, Xu G, Zhang H, Xu C, Zhang W et al. (2023) Morphometric feature description of the proximal ulna based on quantitative measurement: a key consideration for implant design. Surg Radiol Anat 45:215–224. 10.1007/s00276-022-03058-8 36509883

[22] Bonett DG (2002) Sample size requirements for estimating intraclass correlations with desired precision. Stat Med 21:1331–1335. 10.1002/sim.1108 12111881

[23] Mackay ND, Smith NA, Parsons N, Spalding T, Thompson P, Sprowson AP (2014) Medial patellofemoral ligament reconstruction for patellar dislocation: a systematic review. Orthop J Sports Med 2:2325967114544021. 10.1177/2325967114544021 26535352 PMC4555571

[24] Schneider DK, Grawe B, Magnussen RA, Ceasar A, Parikh SN, Wall EJ et al. (2016) Outcomes after isolated medial patellofemoral ligament reconstruction for the treatment of recurrent lateral patellar dislocations: a systematic review and meta-analysis. Am J Sports Med 44:2993–3005. 10.1177/0363546515624673 26872895 PMC5502077

[25] Shah JN, Howard JS, Flanigan DC, Brophy RH, Carey JL, Lattermann C (2012) A systematic review of complications and failures associated with medial patellofemoral ligament reconstruction for recurrent patellar dislocation. Am J Sports Med 40:1916–1923. 10.1177/0363546512442330 22679297 PMC3615712

[26] Berton A, Salvatore G, Nazarian A, Longo UG, Orsi A, Egan J et al. (2023) Combined MPFL reconstruction and tibial tuberosity transfer avoid focal patella overload in the setting of elevated TT–TG distances. Knee Surg Sports Traumatol Arthrosc 31:1771–1780. 10.1007/s00167-022-07056-6 35819464

[27] Lamplot JD, Jahandar A, Meyers KN, Gomoll AH, Maher SA, Strickland SM (2023) Anteromedialization tibial tubercle osteotomy improves patellar contact forces: a cadaveric model of patellofemoral dysplasia. Am J Sports Med 51:453–460. 10.1177/03635465221138287 36453729

[28] Zhang ZJ, Di MLQ, Song GY, Li Y, Cao YW, Zheng T et al. (2023) Clinical and second-look arthroscopic results for derotational distal femoral osteotomy with medial patellofemoral ligament reconstruction for recurrent patellar dislocation with increased femoral anteversion: a series of 102 cases with a minimum clinical follow-up of 2 years. Am J Sports Med 51:663–671. 10.1177/03635465221147484 36661484

[29] Hiemstra LA, Rousseau-Saine A, Lafave MR, Kerslake S (2025) Thin flap trochleoplasty with medial patellofemoral ligament reconstruction for recurrent patellofemoral instability with high-grade trochlear dysplasia: a series of 63 consecutive cases. Am J Sports Med 53:832–838. 10.1177/03635465251314882 39910743

[30] Høj S, Lundegaard JK, Blønd L, Lavard P, Rechter AS, Dippmann C et al. (2025) Open and arthroscopic deepening trochleoplasty improves post-operative outcomes: a systematic review of the literature reveals lack of comparability between techniques. Knee Surg Sports Traumatol Arthrosc. 10.1002/ksa.12647 40095317 PMC12747630

[31] Jud L, Klenecky V, Neopoulos G, Ackermann J, Fucentese SF, Vlachopoulos L (2024) No significant change of tibiofemoral rotation after femoral rotational osteotomy in patients with patellofemoral instability. Knee Surg Sports Traumatol Arthrosc 32:2213–2218. 10.1002/ksa.12250 38713879

[32] Zhang Z, Wang D, Liu Y, Wang X, Zhang H (2025) Short-term outcomes after derotational femoral osteotomy to correct abnormal tibiofemoral rotation in patients with recurrent patellar dislocations and severe rotational malalignment. Am J Sports Med 53:2609–2617. 10.1177/03635465251360785 40815850

[33] Dennis ER, Marmor WA, Pahapill NK, Propp BE, Gruber S, Nguyen JT et al. (2026) Outcomes of medial patellofemoral ligament reconstruction with concomitant tibial tubercle osteotomy for failed surgery for patellar instability versus primary medial patellofemoral ligament reconstruction with concomitant tibial tubercle osteotomy. Am J Sports Med 54:590–601. 10.1177/03635465251409151 41588807

[34] Hiemstra LA, Sheehan B, Sasyniuk TM, Kerslake S (2022) Inter-rater reliability of the classification of the J-sign is inadequate among experts. Clin J Sport Med 32:480–485 10.1097/jsm.0000000000000997 36083327

