# Supplementary material for: Jumping patella is associated with inferior clinical outcomes after MPFL reconstruction for recurrent patellar dislocation: a retrospective cohort study
Source: Knee Surg Relat Res. 2026 Sep 4;38:47. doi: 10.1186/s43019-026-00347-z (PMC13545799; doi:10.1186/s43019-026-00347-z)
Supplement: Supplementary file 1 [file 43019_2026_347_MOESM1_ESM.docx]

**Appendix-1 Classification of the J-sign by the quadrant method**

| **J-sign grade** | **Clinical examination features** |
| --- | --- |
| J- (normal-grade) | patellar displacement not exceeding 1/4 of the maximum transverse diameter of the patella. |
| J1+ (mild) | patellar displacement not exceeding 2/4. |
| J2+ (moderate) | patellar displacement exceeding 2/4. |
| J3+ (high) | Habitual patellar dislocation in extension: complete patellar dislocation occurs with each knee extension. |
