# Supplementary material for: Jumping patella is associated with inferior clinical outcomes after MPFL reconstruction for recurrent patellar dislocation: a retrospective cohort study
Source: Knee Surg Relat Res. 2026 Sep 4;38:47. doi: 10.1186/s43019-026-00347-z (PMC13545799; doi:10.1186/s43019-026-00347-z)
Supplement: Supplementary file 2 [file 43019_2026_347_MOESM3_ESM.docx]

**Appendix 3** Reliability analysis of the graft laxity and bony structure measurement.

| Variables | ICC (interobserver) | 95% Confidence Interval | |
| --- | --- | --- | --- |
|  |  | Lower bound | Upper bound |
| Graft laxity index | 0.88 | 0.82 | 0.93 |
| Femoral torsion | 0.89 | 0.83 | 0.96 |
| Knee torsion | 0.93 | 0.86 | 0.98 |
| Tibial torsion | 0.92 | 0.88 | 0.96 |
| TT-TG | 0.89 | 0.81 | 0.94 |
| TT-PCL | 0.93 | 0.86 | 0.95 |
| CDI | 0.95 | 0.91 | 0.98 |
| Supratrochlear bump | 0.85 | 0.78 | 0.91 |
| LTI | 0.80 | 0.71 | 0.86 |
| Sulcus angle | 0.82 | 0.76 | 0.87 |
| Trochlear depth | 0.81 | 0.74 | 0.89 |

FAA, femoral anteversion angle; TT-TG, tibial tuberosity-trochlear groove distance; TT-PCL, tibial tuberosity-posterior cruciate ligament; CDI, Caton-Deschamps index; LTI, lateral trochlear inclination; ICC, intraclass correlation efficient.
