# Supplementary material for: Jumping patella is associated with inferior clinical outcomes after MPFL reconstruction for recurrent patellar dislocation: a retrospective cohort study
Source: Knee Surg Relat Res. 2026 Sep 4;38:47. doi: 10.1186/s43019-026-00347-z (PMC13545799; doi:10.1186/s43019-026-00347-z)
Supplement: Supplementary file 3 [file 43019_2026_347_MOESM4_ESM.docx]

**Appendix 4** ROC analysis of bony parameters for the preoperative Jumping patella

FAA, femoral anteversion angle; SA, Sulcus angle; TT-TG, tibial tuberosity-trochlear groove distance; TT-PCL, tibial tuberosity-posterior cruciate ligament; CDI, Caton-Deschamps index; LTI, lateral trochlear inclination
